# Network Analysis Reveals A Signaling Regulatory Loop in the *PIK3CA*-mutated Breast Cancer Predicting Survival Outcome

**DOI:** 10.1016/j.gpb.2017.02.002

**Published:** 2017-04-07

**Authors:** Shauna R. McGee, Chabane Tibiche, Mark Trifiro, Edwin Wang

**Affiliations:** 1 National Research Council Canada, Montreal, QC H4P 2R2, Canada; 2 Department of Experimental Medicine, McGill University, Montreal, QC H3A 2B2, Canada; 3 Center for Bioinformatics, McGill University, Montreal, QC H3G 0B1, Canada; 4 Lady Davis Institute for Medical Research of the Jewish General Hospital, Montreal, QC H3T 1E2, Canada; 5 Center for Health Genomics and Informatics, Calgary, AB T2N 4N1, Canada; 6 Department of Biochemistry & Molecular Biology/Medical Genetics/Oncology, University of Calgary Cumming School of Medicine, Calgary, AB T2N 4N1, Canada; 7 Alberta Children's Hospital Research Institute, Calgary, AB T2N 4N1, Canada; 8 Arnie Charbonneau Cancer Research Institute, Calgary, AB T2N 4N1, Canada; 9 O’Brien Institute for Public Health, Calgary, AB T2N 4N1, Canada

**Keywords:** Network analysis, *PIK3CA* mutation, Network motif, Breast cancer, Genome sequencing, Survival

## Abstract

Mutated genes are rarely common even in the same pathological type between cancer patients and as such, it has been very challenging to interpret **genome sequencing** data and difficult to predict clinical outcomes. *PIK3CA* is one of a few genes whose mutations are relatively popular in tumors. For example, more than 46.6% of luminal-A **breast cancer** samples have *PIK3CA* mutated, whereas only 35.5% of all **breast cancer** samples contain ***PIK3CA* mutations**. To understand the function of ***PIK3CA* mutations** in luminal A **breast cancer**, we applied our recently-proposed Cancer Hallmark Network Framework to investigate the **network motifs** in the *PIK3CA*-mutated luminal A tumors. We found that more than 70% of the *PIK3CA*-mutated luminal A tumors contain a positive regulatory loop where a master regulator (PDGF-D), a second regulator (FLT1) and an output node (SHC1) work together. Importantly, we found the luminal A **breast cancer** patients harboring the ***PIK3CA* mutation** and this positive regulatory loop in their tumors have significantly longer **survival** than those harboring ***PIK3CA* mutation** only in their tumors. These findings suggest that the underlying molecular mechanism of ***PIK3CA* mutations** in luminal A patients can participate in a positive regulatory loop, and furthermore the positive regulatory loop (*PDGF-D/FLT1/SHC1*) has a predictive power for the **survival** of the *PIK3CA*-mutated luminal A patients.

## Introduction

Whole-genome and whole-exome sequencing from tumor samples have identified mutations that are enriched in tumor samples in comparison to germline cells. It is widely accepted that these sets of mutations are the causal drivers of tumor progression [1]. However, genetic mutations in tumors are very heterogeneous, meaning that the type and frequency of mutated genes in patients are not only different between cancer types, but also within a same cancer type. The extent of such heterogeneity has recently been demonstrated in genome sequencing of breast cancer samples by The Cancer Genome Atlas (TCGA) [2]. This feature of the cancer mutations makes it challenging to interpret the data and very hard to conduct clinical predictions using genome sequencing data. Interestingly, although mutated genes are rarely common among the breast cancer samples, approximately 29%–45% of all luminal tumor samples harbor *PIK3CA* mutations [2]. In general, breast cancers are classified into three molecular subtypes based on their gene expression profiles: luminal A/B, basal, and HER2 subtypes. The luminal subtypes A/B are often characterized by the expression of estrogen receptor (ER^+^) and represent ∼70% of breast cancer samples [3].

It is now well accepted that cancers do not result from a single mutation or gene, but a combination of perturbed genes acting in molecular networks that correspond to hallmark processes such as apoptosis and cell proliferation [4], [5]. Specifically, mutations in signaling proteins may over-enrich key signaling pathways or inhibit the function of tumor suppressor proteins, both of which can give rise to uncontrolled cell growth and tumor progression [6]. The *PIK3CA* gene, which encodes phosphatidylinositol-4,5-bisphosphate 3-kinase catalytic subunit alpha, is mutated in a number of tumors, including glioblastomas, gastric cancers, lung cancers, ovarian cancers, hepatocellular carcinomas, endometrial carcinomas, brain cancers, and breast cancers [7].The higher frequency of *PIK3CA* missense mutations in luminal breast cancer samples prompted us to ask how *PIK3CA* mutations interact with other mutated genes to trigger cancer progression and metastases. The aim of this study is to identify how *PIK3CA* mutations alter expression of other genes and whether this could be predictive for clinical outcomes.

Cancer mutations have been traditionally investigated in the context of signaling pathways. However, cross-talks frequently occurring among pathways turn the cellular system into a network. Network analysis has provided a simple yet efficient method to model biological systems [8]. In a network, the nodes or vertices of a molecular network represent biomolecules (genes or proteins) while the edges or links represent their physical or functional interactions. An analogy of a roadmap can be used to describe systems biology of the cellular network: if there is car accident on a busy road, drivers will find alternate routes to arrive at their destination. The roadmap provides a collection of intertwined roads and intersections, laid out to visual alternate routes. The cell is organized in the same way––molecules in cells are networked. If a protein in a signaling network is altered, the entire function of a cell could be compromised resulting in a disease phenotype [9].

Cancer signaling often hijack normal human signaling networks and motifs by changing key genomic factors such as gene mutations [9]. Signaling network motifs are a group of interacting proteins acting in the network together and are capable of signal processing. They bear specific regulatory properties and mechanisms as seen in biological network studies [10], [11]. The structure and properties of frequently-occurring network regulatory motifs highlight the functional organization of these signaling networks. By studying the distributions of these network motifs, we can garner insight into cancer-signaling regulatory molecular mechanisms of tumorigenesis and identification of these loops can have practical implications such as prediction of prognosis and the clinical outcomes of cancer patients [12]. For instance, Cui et al. first demonstrated that mutated cancer driver genes are typically enriched in positive network motifs [13], while Fu et al. showed that cancer network motifs are predictive for cancer recurrence [14]. Network modules that are composed of network motifs also play critical roles in cancer progression, metastasis [15], and drug response [16]. Collectively, these studies have demonstrated that network motifs and modules are critical for cancer signaling and associated with clinical outcomes.

The understanding of the cancer hallmarks has represented the most important development in cancer research in the past of 50 years, although these findings are typically descriptive. Based on these results, we developed a network-based approach, Cancer Hallmark Network Framework, to construct predictive models using genome sequencing data [5]. We propose that cancer hallmarks can be represented, quantified, and further modeled computationally, using cancer hallmark networks in an evolutionary context. Different hallmark networks interact with each other during tumor evolution, and furthermore, use distinctive yet complementary capabilities. These capabilities enable tumor growth and metastasis and constitute the organizing principles that provide a logical framework for understanding the diversity of cancer.

In this study, we applied the Cancer Hallmark Network Framework to investigate how *PIK3CA* mutations interact with others in a luminal-breast cancer survival network and ask if the interactions could predict clinical outcomes.

## Results

### *PIK3CA* mutations are significantly higher in luminal A breast cancer subtype

**Figure 1:**
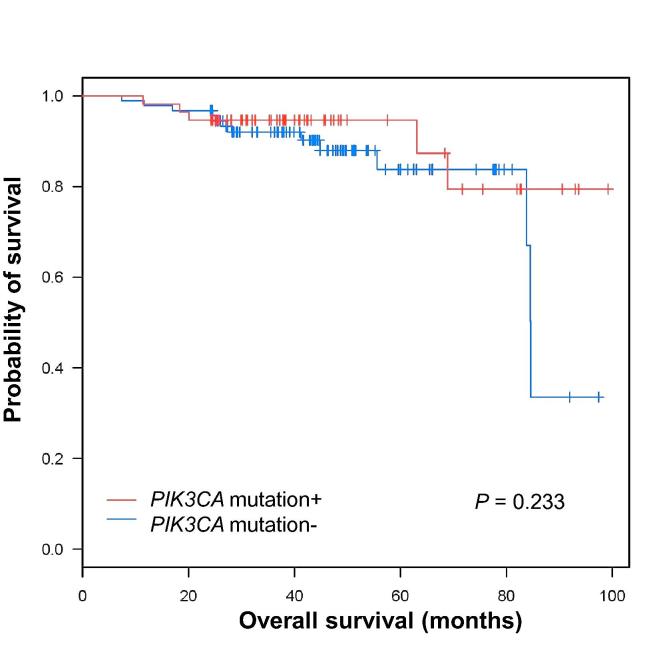
**Kaplan–Meier survival plot for luminal A breast cancer patients with and without *PIK3CA* mutation** Survival plots are shown between luminal A subtype breast tumor patients with (*n* = 137, red) and without (*n* = 211, blue) *PIK3CA* mutation. *P* values were obtained by conducting log-rank test.

A comprehensive analysis of the mutational landscape across 12 major cancer types revealed that *PIK3CA* mutations occurred in 35.5% of all breast cancer tumors, but luminal A subtype tumors had the highest frequency at 46.6% of all the samples harboring the mutation [2]. Thus, we focused on luminal A subtype for further analysis. We obtained whole-exome sequencing data of the tumors from TCGA [2]. Of the 776 breast cancer tumor samples with clinical data available, 358 were luminal subtype and 137 harbored the *PIK3CA* mutation. To test whether the *PIK3CA* mutations correlate with survival in luminal A breast cancer patients, we conducted a survival analysis comparing *PIK3CA*-mutated and *PIK3CA* non-mutated patient tumor samples. There was no significant difference in survival between these two groups (*P* = 0.233, log-rank test) (Figure 1), suggesting that *PIK3CA* mutation alone is not associated with clinical outcomes. Our results suggest that *PIK3CA* mutations interact with other mutated proteins and may play a role in cancer progression or metastasis.

### A Cancer Hallmark Network Framework-based study of cancer genome sequencing data

As mentioned above, mutated genes are rarely shared among breast cancer samples. Therefore, it is challenging to identify meaningful patterns of the mutated gene combinations. Thus, we applied the Cancer Hallmark Network Framework to study the *PIK3CA* mutations in a human signaling network. Cancer cells acquire functional capabilities that allow them to survive, proliferate, disseminate, colonize, and metastasize. It is believed these functions are acquired in different tumors at various time points during tumorigenesis by activating distinct hallmark networks [17]. The core network critical for these processes is the cancer survival network (survival and proliferation) [5]. Within these networks, mutations are not only the master drivers of tumorigenesis but also orchestrate other hallmark capabilities. The evolving cells acquire other hallmark traits based mainly on the activation of the mutation network. In this cellular survival network, mutated genes continually send positive feedback (*i.e.*, selecting the fittest cancer cells) and drive the evolutionary path of these cells. We hypothesized that this positive regulation is controlled by only a handful of genes. They form sub-networks or ‘network motifs’ that frequently recur and consist of functionally-linked molecules, which work together to achieve a distinct function.

**Figure 2:**
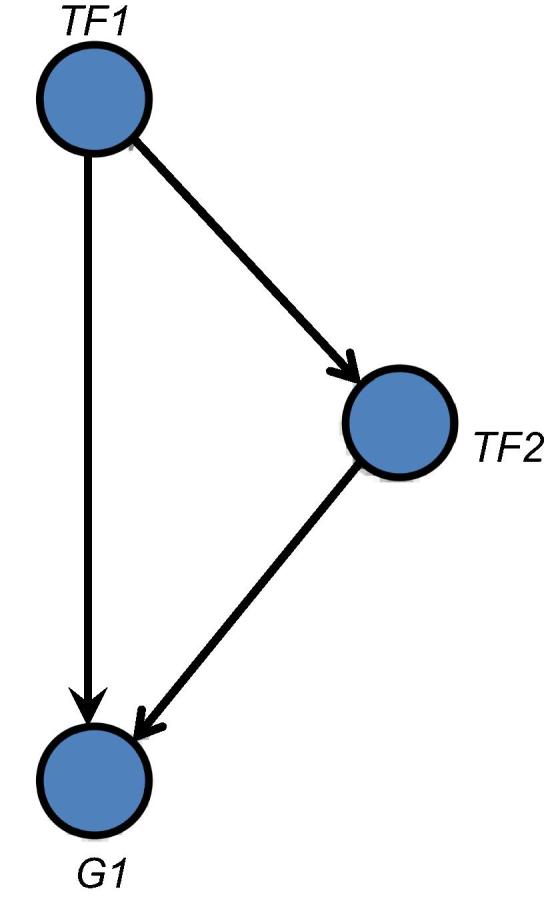
**The diagram of a feed forward loop** Network motifs in a feed forward loop configuration with nodes representing genes and lines and arrows represent gene regulations. Transcription factor (TF1) regulates the second transcription factor (TF2), both of which regulate a target gene (G1).

Biological network motifs drive very specific functions depending on the needs of the cell. Network motifs are subsets of the large, complex network forming significant patterns that reduce the global properties of a network into local functions of network units [18]. Among these motifs, the feed-forward loop (FFL) is a biologically-significant pattern comprised of 3 genes including one target gene and two input genes, one of which regulates the other and jointly regulate a target gene. In terms of regulation, these recurring structural patterns that self-organize to account for the large ratio of genes to transcription factors (TFs) in the genome (Figure 2) [11]. Therefore, we focused on studying the *PIK3CA* mutations and its interactions with other mutated genes in the context of FFL motifs.

**Figure 3:**
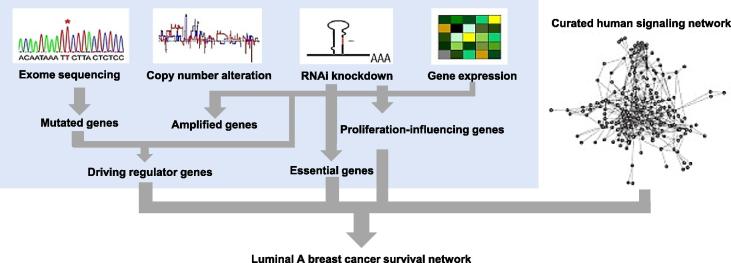
**Survival network construction for luminal A breast cancer** The data from exome sequencing, genome-wide RNAi screening, copy number variations, and gene expression profiles of 5 individual luminal A breast cancer cell lines were examined and genes selected for survival and proliferation. These selected genes were mapped to the human signaling network to create luminal A breast cancer-specific survival network and from this network, all possible regulating feed forward loop configurations were identified.

We first constructed a luminal A-specific survival network. To do so, we applied the same method developed by Zaman et al. [16] and used shRNAi screening of luminal A cell lines (see details in Methods). If a gene is knocked down and the growth of the cancer cell line is reduced, this gene is considered to be associated with survival of this cancer cell line. To create a luminal A breast cancer-specific ‘survival network’, genes determined to be involved in the progression and survival in at least one of the luminal A breast cancer cell lines were mapped onto a human signaling network of known protein interactions. These genes and their links were extracted from this network to form the luminal A-specific ‘survival network’ (see Methods and Figure 3). The original human signaling network of manually-curated interactions consists of over 6000 proteins and 63,000 interactions [6], [9], [19]. The luminal A-specific survival network is generated uniquely by the genes forming a signaling network important for the survival of the tumor.

Because most of the other mutated genes are not common among samples, it is difficult to find patterns of the combinations of the *PIK3CA* mutations with other mutated genes. Based on the Cancer Hallmark Network Framework, we applied a ‘network-profiling’ approach in the survival network. By applying this approach, equal weight can be given to mutations regardless of their frequency, thereby allowing for detection of mutations that may have important biological implications but may not be detected due to their low frequency. Network profiling allows for the examination of the collective effect of genomic alterations, *i.e.*, functionally-mutated genes, on cancer hallmark networks that better represent the phenotypic consequences of mutated genes.

Thus, for each luminal A tumor sample, we applied a network propagation algorithm (see Methods). Briefly, for a given sample, we mapped all the ‘functionally-mutated genes’ (*i.e.*, missense mutations) onto the survival network. These mutated genes are treated as ‘seeds’ and used for the network propagation algorithm. Finally, all the network genes on the survival network had ‘heating scores’. Thus, for that sample, we obtained a network-profile where each network gene had a ‘heating score’. This network profile is similar to the gene expression microarray data. Finally, each luminal A tumor sample has its own unique network-profile based on its mutations. The network profiles of these samples were then normalized (see Methods).

### A FFL harboring ***PIK3CA*** mutations predicts clinical outcomes

Based on the network analysis, a positively regulating FFL is one of the most biologically meaningful network motifs. Therefore, we focused on identifying significant FFL circuits throughout our newly-constructed cancer survival network. The hypothesis is that FFL positively-regulating motifs exist in the subset of luminal A tumor samples harboring the *PIK3CA* mutation that may be involved the cancer process.

**Figure 4:**
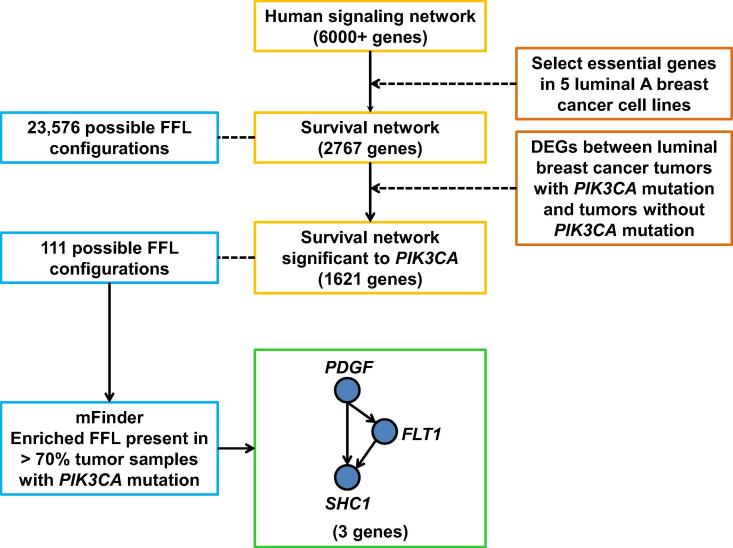
**Workflow diagram to select the positive regulating FFLs** In the luminal A breast cancer survival network, there are 23,576 FFLs. Genes from the survival network were further stratified by selecting only those differentially expressed between luminal A tumors with mutation in *PIK3CA* and tumors without mutation in *PIK3CA*. The resulting network containing 1621 genes yielded 111 possible FFLs. To select FFLs important in *PIK3CA*-mutated tumors, we obtained the network propagation score values for each gene based on mutation status in each tumor and input these data into mFinder. One FFL pattern was found, of which gene interaction ratio was higher than average for each of the 3 nodes in the FFL and enriched in >70 tumor samples. FFL, feed forward loop; DEG, differentially-expressed gene.

First, from the luminal A tumor survival network, we calculated all possible gene combinations in a FFL configuration, resulting in 23,576 FFLs. Second, due to the volume of combinations, we stratified important genes involved in these interactions. We downloaded the tumor RNA-seq expression data from the TCGA for the tumor samples from which the mutations were used in the network. The genes significantly modulated between the *PIK3CA* mutated and non-mutated luminal A tumors were selected, thus resulting in 1621 genes. Only the FFLs containing all the genes that are modulated genes were selected. This restricted the possible FFL configurations to 111 FFLs. We found that among the 111 FFLs, ∼70% of the FFLs of *PIK3CA*-mutated breast luminal A tumors harbor *SHC1*, which encodes the SH2 domain-containing transforming protein 1 (Figure 4). In contrast, this specific FFL was not significantly enriched in any of the non *PIK3CA*-mutated luminal A tumor samples.

It is important to note that in our analysis, if using gene mutation data alone, none of the genes in the positively regulating FFL would have been identified, since the mutation frequency of *PDGF-D*, *FLT1*, and *SHC1* are very low across all breast cancer samples. These results highlight the power of the network-profiling approach and the Cancer Hallmark Network Framework-based approach. These results further suggest that in *PICK3CA*-mutated luminal A tumor samples, a positive FFL consisting of a master regulator (*i.e.*, PDGF-D), a second regulator (*i.e.*, FLT1), and an output node (*i.e.*, SHC1) work together with *PICK3CA* mutations. PDGF-D encodes platelet-derived growth factor D, which is known for its involvement in cell growth, division, and angiogenesis. PDGF-D has been documented as a popular drug target in cancer therapeutics since its receptor is active in the stroma of solid tumors [20]. *FLT1* encodes a member of the vascular endothelial growth factor (VEGF) receptor, which are transmembrane tyrosine kinases involved in angiogenesis and vasculogenesis, suggesting that *FLT1* may be implicated in cancer cell invasion [21]. SHC1 is an adaptor that couples activated growth factors to receptors in signaling pathways. There are three isoforms: p66Shc is involved in cellular lifespan and regulates reactive oxygen species (ROS) production in mitochondrial matrix [22], while the two other isoforms, p52Shc and p46Shc, are linked to tyrosine kinases in the Ras pathway [23].

### The FFL (*PDGF/FLT1/SHC1*) is associated with clinical outcomes

**Figure 5:**
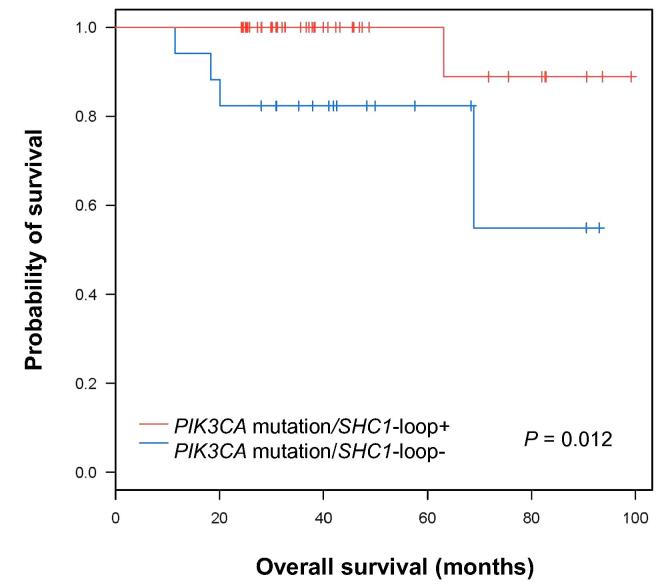
**Kaplan–Meier survival plot for the *PIK3CA*-mutated luminal A breast cancer patients with and without the *SHC1* FFL in their tumors** Survival plots are shown between luminal A subtype breast tumor patients harboring *PIK3CA* driver gene-mutated *SHC1* FFL (*n* = 100, red) and those without the *SHC1* FFL (*n* = 37, blue). *P* values were obtained by conducting log-rank test. FFL, feed forward loop.

To test if the FFL (*PDGF/FLT1/SHC1*) is associated with clinical outcomes, we conducted survival analysis of patients with luminal A breast cancer and *PICK3CA* mutation (Kaplan–Meier plots and log-rank tests) by comparing patients with the FFL (*PDGF/FLT1/SHC1*) (*n* *=* 100) and those without the FFL (*PDGF/FLT1/SHC1*) (*n* *=* 37). Surprisingly, the recurrence rates of the *PIK3CA*-mutantation/*SHC1*-loop+ luminal A tumor patients were lower in the first 5 years after diagnosis than *PIK3CA*-mutantation/*SHC1*-loop^−^ luminal A tumor patients (*P* < 0.01, log-rank test, Figure 5). These results were in stark contrast to those observed between the luminal A tumor patients with *PIK3CA* mutations (*n* *=* 137) and those without *PIK3CA* mutations (*n* *=* 211) (Figure 1). These findings suggest that the luminal A tumor patients harboring the *PIK3CA* mutations and the FFL(*PDGF/FLT1/SHC1*) have significantly longer survival than patients harboring *PIK3CA* mutations in their tumor only.

**Figure 6:**
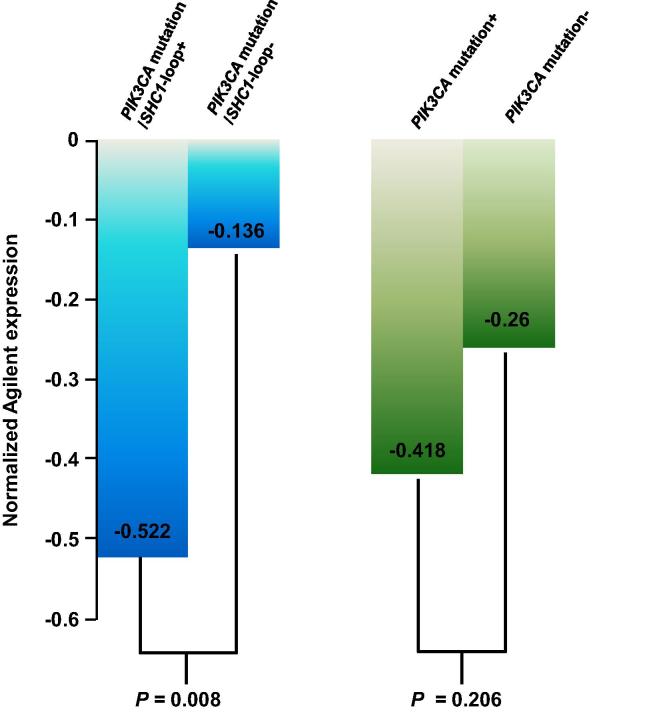
**PAM 50 proliferation index analysis for the luminal A breast cancer patients** Mean PAM50 proliferation index was plotted for gene expression between *PIK3CA*-mutated luminal A breast cancer patients with and without the *SHC1* FFL; and between luminal A breast cancer patients with and without the *PIK3CA* mutation. PAM50 proliferation index comprises 11 proliferation genes that were previously used for predicting tumor progression in breast cancer patients [20]. *P* values were obtained by conducting *t*-test. PAM, Prediction Analysis of Microarrays.

To further validate these results, we applied a proliferation index which is derived from expression values of 11 proliferation genes from the prediction analysis of microarrays (PAM) 50 gene set. PAM50 proliferation index was previously used for predicting tumor progression in breast cancer patients [24], and the predictive value of the PAM50 proliferation score has been demonstrated in other breast cancer patient cohorts as well [25]. We showed that when PAM50 was applied, the average expression of 11 proliferation genes (Figure 6) were significantly lower in the *PIK3CA*-mutated sample cohort harboring the FFL (*PDGF/FLT1/SHC1*) loop than those in the *PIK3CA*-mutated-tumors without the FFL motif (*P* *=* 0.008, *t*-test). In comparison, there were no significant differences in the expression of these 11 genes between tumors with and tumors without the *PIK3CA* mutation. Lower expression of these 11 genes indicate a lower proliferation rate (*i.e.*, longer survival for the patient) of a tumor sample [24]. Therefore, the results of the PAM50 proliferation index are consistent with the results of the FFL motif analysis mentioned above, suggesting that the FFL motif *PDGF/FLT1/SHC1* participates in a critical underlying mechanism for cancer cell proliferation in luminal A *PIK3CA*-mutated tumors.

## Discussion

To date, no algorithms have been developed for constructing predictive models using genome sequencing data. In this study, we applied our recently-proposed Cancer Hallmark Network Framework to investigate the genome mutations on a cancer survival network, one of the key cancer hallmark networks. We showed that FFL (*PDGF/FLT1/SHC1*) is significantly enriched in the *PIK3CA*-mutated luminal A tumor patients, and furthermore, that the luminal A tumor patients harboring the *PIK3CA* mutation and this novel positively regulating loop in their tumors have significantly longer survival than those containing *PIK3CA* mutation only in their tumors. This observation can be obtained only when we applied the cancer hallmark network framework-based approach to the genome sequencing data, indicating that the network and systems approaches are critical in studying the genome sequencing data, either for interpreting the data to gain biological meanings or for constructing predictive models. It is well known that data interpretation and predictive model construction using genome sequencing data have been very challenging until now. The current study demonstrates that the cancer hallmark network framework is a powerful concept and tool for revealing molecular mechanisms and constructing predictive models using genome sequencing data.

The predictive power of the FFL (*PDGF/FLT1/SHC1*) in the *PIK3CA*-mutated luminal-A tumor patients demonstrated in this study could be further validated in other breast cancer cohorts when genome sequencing data for these cohorts are available in the future. Importantly, based on the findings in this study, it could be hypothesized that the FFL network motif in the *PIK3CA*-mutantion/*SHC1*-loop+ samples may induce a protective mechanism, thus incurring a significantly-reduced recurrence rate in the first 5 years after diagnosis. These results were further validated using the PAM 50 proliferation score that showed the tumors harboring the PIK3CA-mutant/*SHC1*-loop+ had significantly slower progression rate.

*SHC1* (*ShcA*) gene encodes three proteins: The ubiquitously-expressed p46 and p52 are derived from alternate translation initiation. p66 is produced through differential promoter usage and experimental data have shown that its levels are highly variable in cancer cells [26]. p66ShcA has been shown to be involved in the breast cancer plasticity by inducing epithelial-to-mesenchymal transition [27]. In contrast, our findings suggest an underlying regulating mechanism through PDGF-D and FLT1, driven by *PIK3CA* mutation in breast tumors.

Small molecule inhibitors have been used to treat cancers by targeting growth factor receptors and therefore these receptors have become attractive therapeutic targets to treat cancer using small molecule inhibitors [28]. FLT1 is the primary receptor driving angiogenesis during cancer progression and anti-FLT1 antibodies have been shown to suppress tumor growth as FLT1 is expressed in both tumor and stromal cells [29]. Therefore, *in vitro* test on breast cancer cells harboring the active FFL (*PDGF/FLT1/SHC1*) using small molecule inhibitor drugs such as sunitinib, which also targets the PDGF receptors, could be an interesting validation experiment in the future.

*PIK3CA* is frequently mutated in cancer and almost every drug company has a clinical trial underway to test inhibitors targeting the PI3K-AKT-mTOR pathway. However to date, responses of solid tumors to PI3K pathway inhibitor monotherapy remain unremarkable with a rapid emergence of drug resistance [30]. A recent study on cell lines has demonstrated that the presence of *PIK3CA* mutations can sensitize cancer cells to mTOR inhibitor, everolimus [31]. This finding could lead to another predictive angle and biological validation in the future.

In summary, taking a systems approach based on the cancer hallmark network framework, we found that the FFL (*PDGF/FLT1/SHC1*) is significantly enriched in the *PIK3CA*-mutated luminal-A tumor patients. More importantly, the luminal-A tumor patients harboring the *PIK3CA* mutation and this positively regulating loop in their tumors have significantly longer survival than those whose tumors containing *PIK3CA* mutation only. Further validation of the FFL (*PDGF/FLT1/SHC1*) as predictive factor for the survival of the *PIK3CA*-mutated luminal-A tumor patients is necessary. More investigation of the molecular mechanisms of the FFL (*PDGF/FLT1/SHC1*) in the *PIK3CA*-mutated luminal-A tumor patients could lead to better predictions and more personalized treatment.

## Materials and methods

### Data collection

We obtained the exome sequencing data for the 5 breast cancer luminal subtype cell lines originally from ATCC (MDAMB453, SKBR3, T47D, MCF7, and ZR751). Microarray and copy number data were downloaded from the Cancer Cell Line Encyclopedia (CCLE) database (http://www.broadinstitute.org/ccle/home). Genome-wide RNAi screening data of cell survival and proliferation for these cell lines were downloaded from the COLT-Cancer database [32]. Breast cancer tumor whole-exome sequencing sample data were retrieved from TCGA.

### Luminal A survival network construction

To construct the network, the essential genes, driving regulators, and proliferation-influencing genes for the luminal A breast cancer were mapped onto the human signaling network and then the nodes and their links were extracted to create a luminal A-specific survival network (Figure 3). Our current manually-curated network is the largest literature-curated human signaling network, containing more than 6000 proteins and 63,000 relations (Version 7, http://www.cancer-systemsbiology.org/HuamnSignalingNet_v7.csv). These gene interactions represent activation, inhibition, and complexes that play roles in cell signaling. The network is updated every year using data from sources include BioCarta, Cell Signaling Technology (CST) Pathways, Pathway Interaction Database (PID), Information Hyperlinked over Proteins (iHOP), and review papers on cell signaling [5]. To obtain the essential genes, driving regulators, and proliferation-influencing genes for the luminal A breast cancer, for the RNAi screening of the entire genome, we obtained the gene activity rank profile (GARP) scores of the 5 luminal A breast cancer cell lines and RNAi-screening *P* values. Details for calculating GARP scores and RNAi-screening *P* values were described previously [33]. If a gene in a cell line has a RNAi-screening *P* value <0.05 and is not a housekeeping gene, we defined it as a “cancer-essential gene”. The *P* value cut off for defining the cancer-essential genes was supported by validation experiments [33]. A “proliferation-influencing gene” was defined as a gene in a given cell line having an RNAi-screening *P* value <0.1 but >0.05. Knocking down a proliferation-influencing gene will not lead to cell death but significantly reduces cell growth and survival. We then determined that an essential gene and a proliferation-influencing gene in these cell lines should be among the top 75% of the expressed genes for that cell line as described previously [34].

Amplified genes were incorporated into the network if they had a GISTIC score >0.3 and were among the top 50% of the expressed genes for that cell line. The GISTIC cut-off of 0.3 is widely used to define gene amplifications [35], [36]. We selected genes that were essential for cell survival if an amplified gene had an RNAi-screening *P* value <0.4, in the assumption that knocking down the driving regulators would affect cell growth and survival. These genes were nominated as ‘driver regulators’ to the cancer cell growth and survival.

The definitions of these terms were based on certain RNAi-screening *P* value cut-offs. The cut-off values were tested by changing the RNAi-screening *P* values for these genes (*P* < 0.03 or 0.03 < *P* < 0.1 or *P* < 0.5 for cancer-essential genes, proliferation-influencing genes, and driving regulators, respectively). The analysis was rerun and the results were similar to those obtained using the original cut-offs.

### Network propagation, motif detection, and proliferation index score

The network profile for each luminal A breast cancer tumor sample was generated from the newly-constructed luminal A survival network. This was done based on the tumor mutational profiles. Each tumor missense mutated gene was projected as seeds onto luminal A survival network. We then applied the network propagation algorithm to obtain diffusion scores of the genes within the network. We applied a scaling factor of 100,000 to the heating scores and then conducted data transformation using the median centering and z-score between sample approach [37]. The network motifs were extracted using the mFinder tool. The detailed description of using the tool has been described previously [13].

PAM50 proliferation score was measured based on differential expression of the 11 proliferation genes, including *MKI67*, *CDC20*, *BIRC5*, *CCNB1*, *CDCA1*, *CEP55*, *KNTC2*, *PTTG1*, *UBE2C*, *RRM2*, and *TYMS*, as previously demonstrated [24].

## Authors’ contributions

SM participated in project design, data extraction and analysis, statistical analysis, and manuscript drafting. CT participated in the programing and statistical analysis. MT helped in drafting manuscript. EW conceived the idea for the study, participated in its design and coordination, and helped to draft the manuscript. All authors read and approved the final manuscript.

## Competing interests

The authors have declared that there are no competing interests.

## References

[1] Carter H., Chen S., Isik L., Tyekucheva S., Velculescu V.E., Kinzler K.W. (2009). Cancer-specific high-throughput annotation of somatic mutations: computational prediction of driver missense mutations. Cancer Res.

[2] Kandoth C., McLellan M.D., Vandin F., Ye K., Niu B., Lu C. (2013). Mutational landscape and significance across 12 major cancer types. Nature.

[3] Komen SG. Molecular subtypes of breast cancer. [Internet] 2014 [cited 2015 Oct 13]. Available from: http://ww5.komen.org/BreastCancer/SubtypesofBreastCancer.html.

[4] Hanahan D., Weinberg R.A. (2000). The hallmarks of cancer. Cell.

[5] Wang E., Zaman N., Mcgee S., Milanese J.S., Masoudi-Nejad A., O’Connor-McCourt M. (2015). Predictive genomics: a cancer hallmark network framework for predicting tumor clinical phenotypes using genome sequencing data. Semin Cancer Biol.

[6] Cui Q., Ma Y., Jaramillo M., Bari H., Awan A., Yang S. (2007). A map of human cancer signaling. Mol Syst Biol.

[7] Samuels Y., Waldman T. (2010). Oncogenic mutations of PIK3CA in human cancers. Curr Top Microbiol Immunol.

[8] Barabási A.L., Oltvai Z.N. (2004). Network biology: understanding the cell’s functional organization. Nat Rev Genet.

[9] Li L., Tibiche C., Fu C., Kaneko T., Moran M.F., Schiller M.R. (2012). The human phosphotyrosine signaling network: evolution and hotspots of hijacking in cancer. Genome Res.

[10] Babu M.M., Luscombe N.M., Aravind L., Gerstein M., Teichmann S.A. (2004). Structure and evolution of transcriptional regulatory networks. Curr Opin Struct Biol.

[11] Wang E., Purisima E. (2005). Network motifs are enriched with transcription factors whose transcripts have short half-lives. Trends Genet.

[12] Cloutier M., Wang E. (2011). Dynamic modeling and analysis of cancer cellular network motifs. Integr Biol (Camb).

[13] Cui Q., Yu Z., Purisima E.O., Wang E. (2006). Principles of microRNA regulation of a human cellular signaling network. Mol Syst Biol.

[14] Fu C., Li J., Wang E. (2009). Signaling network analysis of ubiquitin-mediated proteins suggests correlations between the 26S proteasome and tumor progression. Mol Biosyst.

[15] Paliouras M., Zaman N., Lumbroso R., Kapogeorgakis L., Beitel L.K., Wang E. (2011). Dynamic rewiring of the androgen receptor protein interaction network correlates with prostate cancer clinical outcomes. Integr Biol (Camb).

[16] Zaman N., Li L., Jaramillo M.L., Sun Z., Tibiche C., Banville M. (2015). Signaling network assessment of mutations and copy number variations predict breast cancer subtype-specific drug targets. Cell Rep.

[17] Gao S., Tibiche C., Zou J., Zaman N., Trifiro M., O’Connor-McCourt M. (2016). Identification and construction of combinatory cancer hallmark-based gene signature sets to predict recurrence and chemotherapy benefit in stage II colorectal cancer. JAMA Oncol.

[18] Milo R., Shen-Orr S., Itzkovitz S., Kashtan N., Chklovskii D., Alon U. (2002). Network motifs: simple building blocks of complex networks. Science.

[19] Awan A., Bari H., Yan F., Moksong S., Yang S., Chowdhury S. (2007). Regulatory network motifs and hotspots of cancer genes in a mammalian cellular signalling network. IET Syst Biol.

[20] Heldin C.-H., Heldin C.-H., Westermark B., Andrae J., Gallini R., Betsholtz C. (2013). Targeting the PDGF signaling pathway in tumor treatment. Cell Commun Signal.

[21] Waltenberger J., Claesson-Welsh L., Siegbahn A., Shibuya M., Heldin C.H. (1994). Different signal transduction properties of KDR and Flt1, two receptors for vascular endothelial growth factor. J Biol Chem.

[22] Zheng Y., Zhang C., Croucher D.R., Soliman M.A., St-Denis N., Pasculescu A. (2013). Temporal regulation of EGF signalling networks by the scaffold protein Shc1. Nature.

[23] Neumann-Haefelin E., Qi W., Finkbeiner E., Walz G., Baumeister R., Hertweck M. (2008). SHC-1/p52Shc targets the insulin/IGF-1 and JNK signaling pathways to modulate life span and stress response in *C. elegans*. Genes Dev.

[24] Martín M., Prat A., Rodríguez-Lescure A., Caballero R., Ebbert M.T.W., Munárriz B. (2013). PAM50 proliferation score as a predictor of weekly paclitaxel benefit in breast cancer. Breast Cancer Res Treat.

[25] Nielsen T.O., Parker J.S., Leung S., Voduc D., Ebbert M., Vickery T. (2010). A comparison of PAM50 intrinsic subtyping with immunohistochemistry and clinical prognostic factors in tamoxifen-treated estrogen receptor-positive breast cancer. Clin Cancer Res.

[26] Galimov E.R. (2010). The role of p66shc in oxidative stress and apoptosis. Acta Naturae.

[27] Hudson J., Ha J.R., Sabourin V., Ahn R., La Selva R., Livingstone J. (2014). P66ShcA promotes breast cancer plasticity by inducing an epithelial-to-mesenchymal transition. Mol Cell Biol.

[28] Wu P., Nielsen T.E., Clausen M.H. (2015). FDA-approved small-molecule kinase inhibitors. Trends Pharmacol Sci.

[29] Fischer C., Mazzone M., Jonckx B., Carmeliet P. (2008). FLT1 and its ligands VEGFB and PlGF: drug targets for anti-angiogenic therapy?. Nat Rev Cancer.

[30] Day F.L., Jorissen R.N., Lipton L., Mouradov D., Sakthianandeswaren A., Christie M. (2013). PIK3CA and PTEN gene and exon mutation-specific clinicopathologic and molecular associations in colorectal cancer. Clin Cancer Res.

[31] Di Nicolantonio F., Arena S., Tabernero J., Grosso S., Molinari F., Macarulla T. (2010). Deregulation of the PI3K and KRAS signaling pathways in human cancer cells determines their response to everolimus. J Clin Invest.

[32] Koh J.L.Y., Brown K.R., Sayad A., Kasimer D., Ketela T., Moffat J. (2012). COLT-Cancer: functional genetic screening resource for essential genes in human cancer cell lines. Nucleic Acids Res.

[33] Marcotte R., Brown K.R., Suarez F., Sayad A., Karamboulas K., Krzyzanowski P.M. (2012). Essential gene profiles in breast, pancreatic, and ovarian cancer cells. Cancer Discov.

[34] Forbes S.A., Bindal N., Bamford S., Cole C., Kok C.Y., Beare D. (2011). COSMIC: mining complete cancer genomes in the Catalogue of Somatic Mutations in Cancer. Nucleic Acids Res.

[35] Mermel C.H., Schumacher S.E., Hill B., Meyerson M.L., Beroukhim R., Getz G. (2011). GISTIC2.0 facilitates sensitive and confident localization of the targets of focal somatic copy-number alteration in human cancers. Genome Biol.

[36] Barretina J., Caponigro G., Stransky N., Venkatesan K., Margolin A.A., Kim S. (2012). The Cancer Cell Line Encyclopedia enables predictive modelling of anticancer drug sensitivity. Nature.

[37] Li J., Lenferink A.E.G.G., Deng Y., Collins C., Cui Q., Purisima E.O. (2010). Identification of high-quality cancer prognostic markers and metastasis network modules. Nat Commun.

